# A review of type 2 diabetes mellitus and cognitive impairment

**DOI:** 10.3389/fendo.2025.1624472

**Published:** 2025-08-04

**Authors:** Wen Kan, Meijie Qu, Yunyang Wang, Xianjun Zhang, Lili Xu

**Affiliations:** ^1^ Department of Endocrinology and Metabolic Diseases, Affiliated Hospital of Qingdao University, Qingdao, China; ^2^ Department of Neurology, Affiliated Hospital of Qingdao University, Qingdao, China

**Keywords:** type 2 diabetes mellitus, cognitive impairment, insulin resistance, oxidative stress, mitochondrial dysfunction

## Abstract

The association between type 2 diabetes mellitus (T2DM) and cognitive impairment represents a critical public health concern, particularly against the backdrop of the rising global prevalence of diabetes and aging populations. Accumulating evidence indicates that T2DM is linked to declines in multiple cognitive domains, which may progress to cognitive impairment or even dementia. This cognitive impairment arises from complex interactions among insulin resistance, chronic inflammatory responses, vascular injury and microangiopathy and oxidative stress. Clinical studies suggest that strict glycemic control combined with behavioral and lifestyle interventions may delay cognitive decline, though their long-term efficacy requires further evidence-based validation. Future research should leverage multi-omics technologies to identify early biomarkers for T2DM-related cognitive impairment, elucidate the underlying molecular mechanisms, identify specific therapeutic targets, and develop personalized intervention strategies. This review systematically examines the epidemiological correlations, pathophysiological mechanisms, and advances in clinical management of T2DM-related cognitive disorders, with the aim of providing a theoretical foundation for early prevention and targeted treatment.

## 1 Introduction

T2DM has emerged as a global epidemic and a major public health challenge. Global prevalence of T2DM is expected to increase to 7,079 cases per 100,000 population by 2030, reflecting a sustained upward trend across all regions (1). T2DM significantly elevates the risk of microvascular complications (e.g., retinopathy, nephropathy, neuropathy) and macrovascular diseases (e.g., coronary artery disease, stroke) (2). Cognitive impairment is defined as a decline in one or more cognitive domains, including memory, attention, orientation, judgment, and problem-solving abilities (3). Manifestations of impairment may include slowed information processing, memory deficits, and challenges in learning or decision-making (4). Cognitive impairment severely impacts overall well-being by compromising social engagement and functional independence, increasing reliance on caregivers while reducing autonomy, and posing safety risks, ultimately elevating morbidity and mortality rates through diminished self-care capacity and delayed medical intervention (3, 5). Given the escalating global prevalence of T2DM and its profound implications for cognitive health, elucidating the underlying pathophysiological pathways and identifying preventive interventions are critical for mitigating dual disease burdens. Research in this domain holds significant clinical and societal relevance. Understanding how metabolic dysregulation accelerates neurodegenerative processes could inform early biomarkers for cognitive risk stratification and targeted therapies. Ultimately, advancing this field may enhance patient outcomes and quality of life.

## 2 Pathophysiology of T2DM and its impact on cognitive function

### 2.1 Basic pathogenesis of T2DM

T2DM is characterized by two central pathophysiological defects: pancreatic β-cell dysfunction and insulin resistance (IR). β-cell dysfunction manifests as inadequate insulin secretion or decompensation, ultimately resulting in hyperglycemia. IR refers to diminished responsiveness of peripheral tissues (e.g., skeletal muscle, adipose tissue, liver) to insulin signaling, glucose uptake into these cells becomes inefficient, resulting in its accumulation in the bloodstream and consequent hyperglycemia. To compensate, pancreatic β-cells augment insulin secretion, leading to a state of hyperinsulinemia. These defects mutually reinforce each other, establishing a vicious cycle. Chronic inflammation, ectopic lipid deposition, endoplasmic reticulum stress (ERS), and oxidative stress further exacerbate metabolic dysregulation by impairing insulin sensitivity and/or β-cell function, thereby driving the onset and progression of T2DM and its target organ damage (TOD) (6).

β-cells integrate signals from glucose, free fatty acids (FFA), hormones (e.g., incretins GLP-1/GIP), and neural inputs to dynamically regulate insulin secretion (7). Factors contributing to β-cell failure include: aging and genetic predisposition, incretin resistance/deficiency (e.g., impaired glucagon-like peptide-1 [GLP-1] and glucose-dependent insulinotropic polypeptide [GIP] signaling), lipotoxicity (FFA-induced β-cell apoptosis) and glucotoxicity (chronic hyperglycemia-induced oxidative stress), insulin resistance-induced β-cell stress, islet amyloid deposition (excessive islet amyloid polypeptide [IAPP] aggregation), reactive oxygen species (ROS)-mediated damage and proinflammatory signaling (8).

IR is a multifactorial metabolic disorder marked by reduced responsiveness of insulin-target tissues (liver, adipose tissue, skeletal muscle) to physiological insulin levels. It is closely linked to metabolic syndrome (MS), where obesity, dyslipidemia, and hypertension amplify IR through: proinflammatory cytokine release (e.g., TNF-α, IL-6), ectopic lipid accumulation (hepatic and muscular lipotoxicity), oxidative stress-mediated inhibition of insulin receptor signaling (e.g., PI3K/Akt pathway) (9).

### 2.2 Impact of diabetes-related complications on cognitive function

#### 2.2.1 Cerebrovascular complications

Diabetes-induced macrovascular pathologies, such as atherosclerosis (AS), drive structural alterations in cerebral vasculature, including stenosis, occlusion, and intima-media thickening—hallmarks of AS (10). Chronic vascular occlusion disrupts central hemodynamics, cerebral perfusion, and cerebrovascular function. Key functional impairments include: endothelial-dependent vasodilation deficits and impaired cerebrovascular autoregulation (11, 12). These dysfunctions directly manifest as abnormal cerebral blood flow (CBF) (13). Evidence linking carotid AS to cerebral atrophy suggests that sustained CBF reduction contributes to brain volume loss, a recognized precursor to vascular dementia (VD). Moreover, Chronic hypoperfusion exacerbates neuroinflammation by activating microglia, leading to white matter lesions (WMLs) and neurodegeneration. WMLs are strongly associated with progressive cognitive decline and eventual VD onset.

#### 2.2.2 Diabetic retinopathy

Diabetic retinopathy, a microvascular complication of diabetes, may reflect systemic microvascular damage. Wu et al. reported that DR is associated with psychomotor slowing and attentional deficits. Electrophysiological studies in diabetic models demonstrate reduced neuronal activity in the primary visual cortex, impairing visual information processing (14). Meta-analytic evidence further links retinal pathology to cognitive decline, any retinopathy was significantly associated with the presence of dementia and cognitive deficits, as well as with reduced processing speed (15).These associations persist even in patients without overt retinopathy, suggesting that retinal microvascular changes (e.g., altered vessel caliber, fractal dimension) may serve as biomarkers of cerebral microangiopathy and neurodegeneration. Additionally, diabetes frequently induces visual system dysfunction, including impaired motion detection, reduced contrast sensitivity, and defective color discrimination, which may indirectly exacerbate cognitive load by disrupting sensory input integration (14).

#### 2.2.3 Diabetic kidney disease

Diabetic kidney disease (DKD), a diabetes-specific complication characterized by progressive renal damage, exacerbates blood-brain barrier (BBB) disruption and is increasingly recognized as a risk factor for neurocognitive deficits. Clinical studies reveal a bidirectional relationship between renal dysfunction and cognitive impairment, where elevated urinary albumin excretion rate (UAER) predicts poorer cognitive performance, while declining glomerular filtration rate (GFR) is associated with worsening cognitive scores, highlighting a dose-dependent link between renal impairment and neurodegeneration (16). DKD contributes to cognitive impairment through interconnected pathophysiological pathways. Impaired renal clearance of uremic toxins, such as indoxyl sulfate, promotes neuroinflammation and oxidative stress, accelerating BBB disruption and neuronal apoptosis (17). These mechanisms synergistically disrupt neurovascular homeostasis, creating a self-reinforcing cycle of neurodegeneration and cognitive decline in diabetic patients. Serum creatinine, an indicator of renal dysfunction, inversely correlates with cognitive performance in elderly diabetic populations, suggesting its potential as a prognostic marker for cognitive decline, while early detection of DKD through measures like UAER monitoring can help stratify dementia risk and guide interventions, such as renin-angiotensin system inhibitors, to preserve both renal and cognitive function (18).

#### 2.2.4 Diabetic peripheral neuropathy

Diabetic peripheral neuropathy (DPN), a prevalent complication of diabetes, is increasingly linked to cognitive impairment. Evidence demonstrates that individuals with T2DM and DPN exhibit lower global cognitive performance compared to those without DPN, including deficits in memory, attention, and psychomotor speed (19). In type 1 diabetes, cognitive impairment is similarly pronounced, with DPN patients showing marked impairments in attention, visuospatial/executive functions, language, and abstract reasoning (20). Notably, painful DPN correlates with more severe cognitive deficits than painless DPN (21), particularly in domains of attention and memory. These findings suggest that DPN severity and subtype may serve as biomarkers for neurodegeneration risk in diabetic populations, underscoring the need for integrated neurological and cognitive assessments in diabetes care (22).

#### 2.2.5 Diabetes-related foot complications

Emerging evidence highlights a significant association between diabetes-related foot complications (DRFC) and cognitive impairment. Studies demonstrate that individuals with DRFC exhibit more pronounced cognitive deficits compared to diabetic patients without foot complications, particularly in domains of verbal memory, executive function, and inhibitory control (23). Furthermore, diabetes-associated cognitive impairment encompasses broader deficits, including impairments in attention, memory, executive function, visuospatial abilities, and language. The severity of DRFC-related cognitive decline underscores the need for integrated care models addressing both metabolic and neurological health in diabetic populations (24).

## 3 Mechanistic insights into T2DM and cognitive impairment

### 3.1 Insulin resistance and cerebral metabolism

Insulin acts on insulin receptors in the brain to promote neurotransmitter synthesis, synaptic plasticity, and neuronal differentiation—processes critical for learning and memory. Insulin signaling in the brain is essential for normal cognitive function, particularly in key regions such as the hippocampus and prefrontal cortex (25), where it regulates glucose utilization, energy homeostasis, synaptogenesis and long-term potentiation (LTP). It also governs appetite regulation and systemic metabolic balance (26). Brain insulin resistance (BIR) is recognized as a key factor linking metabolic disorders like obesity and T2DM to cognitive decline. It disrupts normal insulin signaling pathways, leading to mitochondrial structural and functional abnormalities, which in turn result in ​​impaired energy metabolism​​ and ​​increased oxidative stress​​. This dysfunction triggers ​​neuronal damage​​ and ​​cognitive deterioration​​, ultimately contributing to ​​cognitive impairment​​ (27).

BIR reduces plasma levels of neurotrophic factors such as brain-derived neurotrophic factor (BDNF) (28), which plays a critical role in promoting structural and functional plasticity in the central nervous system (CNS) (29). Additionally, BIR downregulates the expression of neuronal glucose transporters (e.g., GLUT4), impairing cerebral glucose uptake (30). This energy deficit results in decreased brain glucose utilization, triggering neuronal metabolic dysfunction. Such metabolic disturbances not only compromise synaptic plasticity but may also threaten neuronal survival, contributing to memory deficits and executive dysfunction.

### 3.2 Chronic inflammatory response

Diabetes mellitus, a chronic metabolic disorder, significantly disrupts systemic and localized inflammatory responses, thereby compromising brain health. The systemic inflammation associated with diabetes promotes increased BBB permeability, neuroinflammation, and subsequent neurodegenerative alterations. These inflammatory processes are mechanistically linked to cognitive impairment and an elevated risk of neurodegenerative disorders, including Alzheimer’s disease and Parkinson’s disease.

Characterized by chronic low-grade inflammation, diabetes alters the expression of key inflammatory mediators such as monocyte chemoattractant protein-1 (MCP-1), interleukin-6 (IL-6), and C-C motif chemokine receptor 2 (CCR2), which collectively modulate peripheral and central immune responses (31). Activation of inflammatory pathways, particularly those mediated by NF-κB, synergizes with oxidative stress and impaired autophagy to exacerbate neuronal damage and accelerate cognitive decline (32). Dysregulated inflammatory signaling in diabetic individuals amplifies ischemic brain injury.

### 3.3 Vascular injury and microangiopathy

Diabetes exerts profound detrimental effects on cerebrovascular health, precipitating vascular injury and microvascular pathologies closely associated with cognitive impairment. The relationship between diabetes and cerebrovascular pathology is multifaceted, encompassing both macrovascular and microvascular abnormalities that synergistically contribute to cognitive decline (33).

Accumulating evidence indicates that diabetes independently increases the incidence of cerebral infarctions and vascular brain injuries, both of which are critical determinants of cognitive deterioration (33). Diabetic-induced cerebrovascular damage manifests as endothelial dysfunction, increased arterial stiffness, and thickening of capillary basement membranes (34). These pathological alterations compromise cerebrovascular elasticity and integrity, leading to reduced cerebral blood flow and chronic cerebral hypoperfusion, thereby exacerbating cognitive deficits.

As an established risk factor for cerebrovascular diseases, diabetes is characterized by a higher prevalence of cerebral infarctions, white matter hyperintensities (WMH), and cerebral small vessel disease (CSVD) (33). These pathologies exhibit greater severity in diabetic populations and correlate with an elevated risk of accelerated cognitive decline. Preclinical studies utilizing diabetic rodent models demonstrate that hyperglycemia exacerbates cerebrovascular dysfunction, which parallels observed cognitive impairments, highlighting diabetes-specific vulnerabilities in neurovascular coupling (35).

### 3.4 Oxidative stress and neuronal injury

Chronic hyperglycemia drives oxidative stress, a pivotal mechanism underlying neuronal injury in diabetes. Sustained elevation of blood glucose promotes excessive generation of ROS and reactive nitrogen species (RNS), overwhelming endogenous antioxidant defenses. This redox imbalance critically contributes multifaceted biochemical cascades and cellular dysfunction. Prolonged hyperglycemia activates four major oxidative stress-inducing pathways: the polyol pathway, advanced glycation end-product (AGE) accumulation, protein kinase C (PKC) activation, and dysregulation of the hexosamine pathway (36, 37). These interconnected pathways work synergistically to amplify ROS production, which directly damages neuronal membranes, proteins, and DNA.

Moreover, oxidative stress activates redox-sensitive transcription factors, such as NF-κB, leading to the upregulation of pro-inflammatory cytokines (e.g., TNF-α, IL-1β, IL-6) (37, 38). These cytokines exacerbate neuroinflammation, creating a vicious cycle in which oxidative stress and inflammation synergistically drive neurodegeneration. Diabetic rodents exhibit elevated oxidative markers (8-OHdG, nitrotyrosine) and increased apoptosis in dorsal root ganglion (DRG) neurons, confirming oxidative damage as a driver of peripheral neuropathy (39).

### 3.5 The interplay between T2DM and Alzheimer’s disease

Emerging evidence reveals significant pathophysiological intersections between diabetes mellitus, particularly T2DM, and neurodegenerative disorders, with Alzheimer’s disease (AD) demonstrating particularly strong associations (40). T2DM has been established as an independent risk factor for accelerated cognitive decline and neurodegeneration, potentially increasing AD susceptibility through shared biological mechanisms. The disease continuum appears driven by overlapping pathways including chronic insulin resistance, systemic inflammation, and mitochondrial dysfunction, which collectively create a neurodegenerative milieu (41, 42). Notably, hallmark AD pathologies – β-amyloid (Aβ) plaque deposition and tau protein hyperphosphorylation – are exacerbated by diabetic metabolic disturbances. Sustained hyperglycemia and impaired insulin signaling in T2DM patients potentiate amyloidogenic processing while compromising tau protein homeostasis, thereby accelerating the neuropathological cascade characteristic of AD progression (43).

### 3.6 T2DM-related cognitive impairment and gut-brain axis disruption

The gut-brain axis is a bidirectional communication system between the gastrointestinal tract and the central nervous system that influences cognitive function through various mechanisms (44). In T2DM, significant alterations occur in the composition of the gut microbiota, which affect cognitive abilities. Specific genera (e.g., *Parabacteroides* and *Lactobacillus*) have been shown to modulate metabolites, thereby improving cognitive behavior in diabetic models (45). Changes in the gut microbiome directly or indirectly impact brain function through vagal, endocrine, and immune pathways. Metabolites directly activate vagal afferent nerves, transmitting signals to the brain via vagal pathways. In the endocrine pathway, enteroendocrine L cells release gut hormones such as GLP1, peptide YY (PYY), and cholecystokinin (CCK), which subsequently influence learning, memory, and mood. Within the immune pathway, immune cells including helper T cell 1 (Th1), helper T cell 17 (Th17), regulatory T cells (Treg), neutrophils, and macrophages are stimulated (46). Fecal microbiota transplantation has demonstrated potential in alleviating diabetes-related cognitive decline by altering gut microbiota composition and enhancing brain insulin signaling pathways (45).

Although the gut-brain axis plays a significant role in T2DM-related cognitive impairment, it is essential to consider other contributing factors, such as genetic predisposition and lifestyle. The interactions between these factors and the gut-brain axis are complex and require further research to fully elucidate the underlying mechanisms.

## 4 Sociodemographic moderators in T2DM-related cognitive impairment

In recent years, an increasing number of studies have explored the potential roles of gender, age, educational level, and social determinants in the link between T2DM and cognitive function from demographic and sociological perspectives, offering new pathways for identifying vulnerable populations and developing intervention strategies.

Age is a key factor for cognitive impairment in T2DM patients. Older age has consistently been associated with a higher risk of cognitive impairment. Studies show that individuals aged 60 and above with T2DM are particularly susceptible to cognitive decline, with the risk increasing with advancing age (47, 48).

Research shows that, independent of diabetes status, women generally perform better in certain cognitive domains (e.g., global cognition and verbal abilities) (49). However, other studies suggest that middle-aged men with T2D have a higher risk of mild cognitive impairment (MCI) compared to women (50).

Education is widely recognized as a core component of cognitive reserve and also plays a significant moderating role in the association between T2DM and cognitive function. Research by Reinke published in *BMC Public Health* indicates that higher education is not only associated with better glycemic control but can also slow diabetes-related cognitive decline (51). Peña-González further points out that education can mitigate the negative effects of the disease by building cognitive reserve, enhancing an individual’s neuroplasticity and cognitive resilience in the face of hyperglycemia or metabolic dysregulation (52).

Social determinants such as socioeconomic status, race/ethnicity, and healthcare access also play crucial roles in T2DM-related cognitive impairment (53). Studies comparing elderly populations in the US and Mexico found that low socioeconomic status is significantly associated with cognitive decline, and the level of social support significantly influences the cognitive trajectory of diabetes (54).

In summary, gender, age, education, and social determinants all play complex and interacting roles in the link between T2DM and cognitive function. Research indicates that individuals with early-onset diabetes, those with lower educational attainment, and those of low socioeconomic status face a higher risk of cognitive impairment. Theories such as cognitive reserve, the accumulated stress model, and research on health inequalities provide multidimensional perspectives for understanding diabetes-induced cognitive impairment. Future research should deepen efforts in the following areas: First, strengthen stratified studies on different population subgroups to precisely identify high-risk groups. Second, integrate metabolic, physiological, psychological, and social dimensions to construct multi-level assessment models. Third, enhance educational interventions and social support within community health promotion to mitigate the cognitive risks stemming from structural health inequalities.

## 5 T2DM management and cognitive impairment

### 5.1 Glycemic control and cognitive function in T2DM

The relationship between glycemic control and cognitive performance represents a critical focus in diabetes research, particularly in the context of neurodegeneration. Accumulating evidence indicates that suboptimal glycemic management is strongly associated with accelerated cognitive impairment, whereas rigorous blood glucose regulation may attenuate neurodegenerative progression. In patients with T2DM, chronic hyperglycemia and elevated glycated hemoglobin (HbA1c) levels correlate with measurable declines in global cognitive function. Multiple studies have shown that improving blood sugar control can enhance cognitive function. For example, a systematic review found that antidiabetic treatment can significantly alleviate the decline in cognitive ability in diabetic patients (55).

### 5.2 Potential effects of common glucose-lowering drugs on cognitive function

The potential impact of glucose-lowering drugs on cognitive function is multifaceted, encompassing both adverse effects resulting from drug-induced hypoglycemia and possible cognitive benefits associated with specific drugs. These effects are systematically summarized in **Table 1**.

**Table 1:** Potential effects of common glucose-lowering drugs on cognitive function.

Medicine	Glucose-lowering efficacy	Core mechanisms of glucose lowering	Cognitive outcomes
Metformin	High	Suppression of hepatic gluconeogenesis and improvement of peripheral insulin sensitivity	Activation of AMP-activated protein kinase (AMPK) signaling and Attenuation of oxidative stress
GLP-1RA	High to very high	Stimulation of insulin secretion and Suppression of glucagon secretion	Reduction of neuroinflammation, enhancement of mitochondrial function, antioxidant stress, prevention of β-amyloid and tau protein aggregation, and improvement of synaptic function
SGLT2is	Intermediate to high	Inhibition of renal glucose reabsorption	Anti-inflammatory actions, antioxidant properties, and modulation of neurotrophic factors
DPP-4i	Intermediate	Inhibition of dipeptidyl peptidase-4 (DPP-4) activity and Enhancement of intact GLP-1 and GIP levels	Activation of AMPK signaling cascade and attenuation of tau protein phosphorylation
Sulfonylureas	High	Potentiation of pancreatic β-cell insulin secretion	Antioxidant stress and promotion of BDNF transcription

As a first-line oral hypoglycemic agent for T2DM, metformin demonstrates bidirectional regulatory effects on cognition. Accumulating evidence suggests its neuroprotective properties through mechanisms including AMPK signaling pathway activation and oxidative stress inhibition, with demonstrated improvements in specific cognitive domains such as verbal learning, working memory, and executive function in diabetic patients (56). Notably, prolonged metformin therapy may induce vitamin B12 deficiency, which has been established as a risk factor for cognitive decline (57, 58). This dual mechanism indicates that while metformin may mitigate diabetes-related neurodegenerative processes, it could paradoxically impair cognitive health in susceptible individuals through nutritional metabolic disturbances. Clinical protocols should therefore incorporate regular serum vitamin B12 monitoring and timely nutritional supplementation to prevent associated complications (59).

#### 5.2.1 GLP-1 receptor agonists

The neuroprotective effects of GLP-1RA are believed to be mediated through multiple mechanisms, including the reduction of neuroinflammation, enhancement of mitochondrial function, antioxidant stress, prevention of β-amyloid and tau protein aggregation, and improvement of synaptic function (60). A systematic review and meta-analysis found that GLP-1RA did not significantly affect overall cognitive function in patients with type 2 diabetes. However, subgroup analysis indicated potential benefits for patients under 65 years of age or those without a history of cardiovascular or cerebrovascular disease, suggesting that age and comorbidities may influence cognitive outcomes with GLP-1 therapy (61). Another study reported that the GLP-1RA liraglutide improved short-term memory and memory composite scores in obese patients with prediabetes or early-stage type 2 diabetes, independent of weight loss effects. This suggests that GLP-1RA provide direct cognitive benefits in early-stage diabetes (62). In animal models, GLP-1RA have shown promising results in improving cognitive function. For example, liraglutide improved memory retention and increased hippocampal neuron count in an Alzheimer’s disease mouse model, indicating its potential neuroprotective effects (63).

#### 5.2.2 Sodium-glucose cotransporter 2 inhibitors

(SGLT2is) exerts beneficial effects on cognitive impairment through multifaceted mechanisms involving metabolic regulation, anti-inflammatory actions, antioxidant properties, and modulation of neurotrophic factors (64). Preclinical studies demonstrate that dapagliflozin ameliorates cognitive deficits in diabetic murine models by restoring hippocampal mitochondrial dynamics - a critical process for synaptic plasticity and memory formation (65). Particularly in dual pathology models combining T2DM and AD, SGLT2is not only enhance insulin sensitivity but also significantly reduce β-amyloid deposition, addressing two fundamental pathological hallmarks of AD.

Clinical investigations reveal substantial cognitive improvements associated with SGLT2i therapy, especially in populations with mild cognitive impairment or established dementia. A meta-analysis quantifying cognitive outcomes showed a standardized mean difference of 0.88 (95% CI: 0.32-1.44) in cognitive assessment scores, indicating clinically meaningful enhancement (66).

Notably, large-scale clinical data analysis demonstrates significant dementia risk reduction with SGLT2i use. In a population-based study of 331,908 T2DM patients, SGLT2i treatment was associated with 23% lower dementia incidence (HR: 0.77; 95% CI: 0.71-0.84) (67). The observed cerebroprotection may derive from synergistic effects including blood pressure optimization, sodium homeostasis regulation, and metabolic reprogramming, collectively preserving neuronal integrity in chronic metabolic disorders (68).

#### 5.2.3 DPP-4 inhibitors

DPP-4is improve glucose tolerance and insulin sensitivity, which are critical for maintaining cognitive function in diabetic patients. Improvements in metabolic parameters such as reduced fasting blood glucose and HbA1c levels are consistently associated with better cognitive outcomes (69, 70). Sitagliptin has been shown to reduce oxidative stress and promote hippocampal neurogenesis, both essential for cognitive health. These effects are further supported by the upregulation of genes involved in neuroprotection and synaptic plasticity (69).

At the molecular level, DPP-4is likely exert their benefits via the AMPK/mTOR pathway, enhancing neuronal autophagy and reducing tau protein phosphorylation—mechanisms that are neuroprotective (71). A meta-analysis involving 5,583 participants demonstrated that DPP-4is significantly reduce the incidence of cognitive impairment in T2DM patients, with a standardized mean difference (SMD) of 0.99. Additionally, clinical studies revealed that these agents improve scores on the Mini-Mental State Examination (MMSE) and Instrumental Activities of Daily Living (IADL), indicating measurable cognitive benefits (70, 72). Notably, in elderly patients with mild cognitive impairment, DPP-4is stabilize cognitive function by reducing glycemic variability (73).

Preclinical evidence further supports these findings: in insulin-resistant rat models, DPP-4is such as sitagliptin and vildagliptin improve cognitive behaviors and restore cerebral mitochondrial function, demonstrating protective effects against diet-induced cognitive decline (74).

#### 5.2.4 Sulfonylureas

Sulfonylureas may exert dual effects on cognitive function in diabetic populations. Preclinical studies indicate potential neuroprotective properties: glimepiride ameliorated learning and memory deficits in diabetic rodent models, potentially through antioxidant mechanisms and upregulation of BDNF levels (75). Similarly, glyburide prevented chronic stress-induced cognitive impairment in murine models, possibly via modulation of the hypothalamic-pituitary-adrenal (HPA) axis and glucocorticoid receptor stabilization (76).

However, these potential benefits must be weighed against well-documented adverse effects. Sulfonylureas are associated with hypoglycemia episodes and progressive β-cell exhaustion, factors that may exacerbate cognitive decline, particularly in elderly patients with compromised cerebral autoregulation. Population-based studies reveal concerning epidemiological patterns. A retrospective cohort analysis (n = 45,632 T2DM patients) comparing sulfonylureas with DPP-4is demonstrated increased dementia risk with sulfonylurea use over 4.82 mean follow-up years (77). Network meta-analyses further position sulfonylureas as second-highest among antidiabetic agents for dementia risk escalation (surface under cumulative ranking [SUCRA] score: 73.4), surpassed only by insulin analogs (78).

### 5.3 Behavioral and lifestyle interventions: synergistic effects of diet, exercise, and cognitive training

Accumulating evidence supports behavioral and lifestyle interventions—including dietary modification, physical exercise, and cognitive training—as promising non-pharmacological strategies to mitigate diabetes-related cognitive decline. T2DM is a well-established independent risk factor for mild cognitive impairment and dementia progression. While current pharmacological interventions remain suboptimal, multimodal lifestyle approaches demonstrate clinically meaningful potential for preserving neurocognitive function in T2DM populations.

#### 5.3.1 Exercise and cognitive function

Controlled trials reveal differential cognitive benefits of structured exercise regimens in T2DM patients. Both resistance training and aerobic exercise protocols show efficacy (79), with meta-analytic data indicating significant attenuation of cognitive decline through multicomponent programs (sessions >40 minutes, ≥4 times weekly over 6 months; pooled effect size: 0.61, 95% CI: 0.40–0.82) (80). Notably, intervention heterogeneity exists, with some studies reporting only moderate effects or null findings in single-modality approaches, underscoring the need for optimized intensity-duration parameters (81, 82).

#### 5.3.2 Cognitive training interventions

Domain-specific cognitive interventions yield measurable neuropsychological improvements. Dual-task training and targeted cognitive remediation significantly enhance memory performance and executive function in T2DM cohorts. Although mechanistic links to glycemic control remain inconclusive, these interventions demonstrate synergistic benefits when combined with lifestyle education, enhancing both cognitive capacity and diabetes self-management skills (83).

#### 5.3.3 Combined interventions

Integrating diet, exercise, and cognitive training—seem to offer the most comprehensive benefits. Research indicates that multimodal interventions can improve cognitive function and overall health outcomes in patients with T2DM (80). The LIFE study, which included physical activity interventions, reported beneficial effects on global cognitive function and memory slowing in elderly individuals with T2DM, highlighting the potential of a combined approach (84).

Current evidence positions multimodal lifestyle interventions as clinically actionable strategies for addressing diabetes-associated cognitive impairment. The triad of dietary optimization, structured exercise, and cognitive engagement demonstrates synergistic neuroprotection, potentially delaying dementia onset while improving metabolic health. Future research should prioritize protocol standardization.

## 6 Biomarkers of cognitive impairment in T2DM

Given the significantly increased risk of dementia and cognitive decline in patients with T2DM, research on predictive biomarkers and early detection technologies for cognitive impairment in this population has gained increasing attention in recent years, which is of great significance for achieving early diagnosis and timely intervention. Recent research advances have revealed several categories of biomarkers with potential clinical applications, including but not limited to Neuron-Derived Exosomes (NDEs) and metabolic signatures.

NDEs are small extracellular vesicles that can cross the blood-brain barrier and are enriched with neuron-specific proteins, RNAs, and other biomolecules. These vesicles have emerged as promising tools for early diagnosis of neurodegenerative diseases (85). Chi et al. discovered that plasma NDEs from T2DM patients showed significantly reduced levels of mitochondrial complex I protein NDUFS3 and complex II protein SDHB. Lower SDHB levels were positively correlated with progression from MCI to AD (86). Specific miRNAs and proteins in NDEs, including but not limited to miR-185-5p, miR-132-3p, and miR-34a-5p (87–89), have been linked to AD and cognitive impairment, highlighting their biomarker potential.

Elevated myo-inositol (MI) concentrations coupled with reduced glutamate (Glu), Glx (glutamate + glutamine), and NAA/Cr (N-acetylaspartate/creatine) ratios within the hippocampus represent sensitive metabolic biomarkers for monitoring diabetic cognitive impairment progression (90). Additional metabolic biomarkers under consideration include C-reactive protein, tau protein, BDNF and AGEs (91).

The hippocampus, a brain region critically involved in learning and memory processes, may serve as an early “warning indicator” for diabetes-associated cognitive impairment through its volumetric changes (92). High-resolution MRI segmentation analyses have demonstrated significant volumetric reductions in memory-critical hippocampal subfields (CA1, dentate gyrus [DG], and subiculum) among middle-aged T2DM patients. These specific subregions are known to be fundamentally involved in memory encoding and consolidation processes. These structurally measured hippocampal subfield atrophies not only directly correlated with cognitive performance but may also reflect persistent neural damage caused by glucose metabolic dysregulation. Importantly, these findings provide neuroimaging evidence for early identification of high-risk populations (93).

In newly diagnosed T2DM patients, elevated venular tortuosity shows significant association with cognitive impairment. This correlation remained significant after adjustment for multiple demographic and health factors, suggesting retinal vascular tortuosity may serve as an early indicator of cognitive decline (94). Retinal vascular metrics (RVMs) can function as imaging biomarkers to predict cognitive impairment in T2DM, thereby enhancing risk stratification (95). Furthermore, patients with T2DM and mild cognitive impairment (MCI) exhibit significant thinning of the macular retinal nerve fiber layer (RNFL) and ganglion cell layer (GCL) (96). These structural changes can be effectively quantified through *in vivo* optical coherence tomography (OCT) imaging, highlighting the potential of retinal assessment for predicting diabetes-related cognitive dysfunction (97).

The complexity of T2DM-related cognitive dysfunction involves multiple pathways and contributing factors, necessitating a multi-faceted approach to biomarker discovery. Future research should prioritize validation of these biomarkers in larger longitudinal cohorts and explore their potential for guiding personalized therapeutic strategies.

## 7 Conclusion

In summary, a complex pathophysiological relationship exists between T2DM and cognitive impairment. Mechanisms including insulin resistance, chronic inflammation, vascular injury, and oxidative stress collectively drive disease progression. Effective glycemic control not only mitigates metabolic dysregulation but also exerts protective effects on cognitive function. Concurrently, factors such as age, educational attainment, and socioeconomic determinants play pivotal roles in disease development, thereby providing new perspectives for multidimensional intervention strategies. Furthermore, the discovery of novel biomarkers and early detection technologies offers promising avenues for early screening and disease monitoring.

These findings hold significant implications for both clinical practice and public health: On the one hand, they underscore the necessity for early cognitive assessment and integrated management in T2DM patients; On the other hand, they emphasize the importance of establishing social support systems to reduce cognitive risk in high-risk populations. However, current research still has limitations, such as small sample sizes in longitudinal cohort studies, incomplete understanding of mechanistic differences across diverse ethnic groups, and the need for further validation of newly identified biomarkers in clinical translation.

Future research could focus on leveraging multi-omics technologies to explore novel pathogenic mechanisms, conducting long-term, multicenter, cross-cultural cohort studies to clarify the universality of risk factors, and developing early warning models based on emerging biomarkers. There is also an urgent need to explore personalized comprehensive treatment strategies that integrate metabolic regulation and social interventions, thereby providing a more robust theoretical and practical foundation for improving cognitive health in patients with T2DM.

## References

[1] Khan MAB Hashim MJ King JK Govender RD Mustafa H AlKaabi J. Epidemiology of type 2 diabetes - global burden of disease and forecasted trends. J Epidemiol Global Health. (2020) 10:107–11. doi: 10.2991/jegh.k.191028.001, PMID: 32175717 PMC7310804

[2] Młynarska E Czarnik W Dzieża N Jędraszak W Majchrowicz G Prusinowski F. Type 2 diabetes mellitus: new pathogenetic mechanisms, treatment and the most important complications. Int J Mol Sci. (2025) 26(3):1094. doi: 10.3390/ijms26031094, PMID: 39940862 PMC11817707

[3] Chang HJ Burke AE Glass RM. Older drivers and cognitive impairment. JAMA. (2010) 303:1660–. doi: 10.1001/jama.281.16.1560 20424258

[4] Shang H Xu C Lu H Zhang J. The early stage of abnormal aging: cognitive impairment. Adv Exp Med Biol. (2023) 1419:149–55. doi: 10.1007/978-981-99-1627-6_11, PMID: 37418212

[5] Foreman MD Grabowski R. Diagnostic dilemma: cognitive impairment in the elderly. J gerontological Nurs. (1992) 18:5–12. doi: 10.3928/0098-9134-19920901-04, PMID: 1430903

[6] Lu X Xie Q Pan X Zhang R Zhang X Peng G. Type 2 diabetes mellitus in adults: pathogenesis, prevention and therapy. Signal transduction targeted Ther. (2024) 9:262. doi: 10.1038/s41392-024-01951-9, PMID: 39353925 PMC11445387

[7] DeFronzo RA Ferrannini E Groop L Henry RR Herman WH Holst JJ. Type 2 diabetes mellitus. Nat Rev Dis Primers. (2015) 1:15019. doi: 10.1038/nrdp.2015.19, PMID: 27189025

[8] Galicia-Garcia U Benito-Vicente A Jebari S Larrea-Sebal A Siddiqi H Uribe KB. Pathophysiology of type 2 diabetes mellitus. Int J Mol Sci. (2020) 21(17):6275. doi: 10.3390/ijms21176275, PMID: 32872570 PMC7503727

[9] Lee SH Park SY Choi CS. Insulin resistance: from mechanisms to therapeutic strategies. Diabetes Metab J. (2022) 46:15–37. doi: 10.4093/dmj.2021.0280, PMID: 34965646 PMC8831809

[10] Alhusaini S Karama S Nguyen TV Thiel A Bernhardt BC Cox SR. Association between carotid atheroma and cerebral cortex structure at age 73 years. Ann Neurol. (2018) 84:576–87. doi: 10.1002/ana.25324, PMID: 30179274 PMC6328248

[11] Aparicio HJ Petrea RE Massaro JM Manning WJ Oyama-Manabe N Beiser AS. Association of descending thoracic aortic plaque with brain atrophy and white matter hyperintensities: The Framingham Heart Study. Atherosclerosis. (2017) 265:305–11. doi: 10.1016/j.atherosclerosis.2017.06.919, PMID: 28673480 PMC5617776

[12] Ogoh S. Relationship between cognitive function and regulation of cerebral blood flow. J Physiol sciences: JPS. (2017) 67:345–51. doi: 10.1007/s12576-017-0525-0, PMID: 28155036 PMC10717011

[13] Moroni F Ammirati E Magnoni M D'Ascenzo F Anselmino M Anzalone N. Carotid atherosclerosis, silent ischemic brain damage and brain atrophy: A systematic review and meta-analysis. Int J Cardiol. (2016) 223:681–7. doi: 10.1016/j.ijcard.2016.08.234, PMID: 27568989

[14] Chen H Wang M Xia L Dong J Xu G Wang Z. New evidence of central nervous system damage in diabetes: impairment of fine visual discrimination. Diabetes. (2022) 71:1772–84. doi: 10.2337/db21-0715, PMID: 35612428

[15] Heringa SM Bouvy WH VanDenBerg E Moll AC Kappelle LJ Biessels GJ. Associations between retinal microvascular changes and dementia, cognitive functioning, and brain imaging abnormalities: a systematic review. J Cereb Blood Flow Metab. (2013) 33:983–95. doi: 10.1038/jcbfm.2013.58, PMID: 23591648 PMC3705441

[16] Shi X Zhang Y Niu H Wang R Shen J Zhou S. Correlation between cognitive impairment and diabetic nephropathy in patients with Type 2 diabetes mellitus. Zhong nan da xue xue bao Yi xue ban = J Cent South Univ Med Sci. (2016) 41:143–50. doi: 10.11817/j.issn.1672-7347.2016.02.005, PMID: 26932211

[17] Bossola M Picconi B. Uremic toxins and the brain in chronic kidney disease. J Nephrol. (2024) 37:1391–5. doi: 10.1007/s40620-024-01929-4, PMID: 38625502

[18] Xiao Y Devakumar V Xu L Liu L Mo H Hong X. Elevated serum creatinine levels and risk of cognitive impairment in older adults with diabetes: a NHANES study from 2011–2014. Front Endocrinol. (2023) 14:1149084. doi: 10.3389/fendo.2023.1149084, PMID: 37900140 PMC10603184

[19] Palomo-Osuna J DeSola H Dueñas M Moral-Munoz JA Failde I. Cognitive function in diabetic persons with peripheral neuropathy: a systematic review and meta-analysis. Expert Rev Neurother. (2022) 22:269–81. doi: 10.1080/14737175.2022.2048649, PMID: 35232335

[20] Ding X Fang C Li X Cao YJ Zhang QL Huang Y. Type 1 diabetes-associated cognitive impairment and diabetic peripheral neuropathy in Chinese adults: results from a prospective cross-sectional study. BMC endocrine Disord. (2019) 19:34. doi: 10.1186/s12902-019-0359-2, PMID: 30917808 PMC6437981

[21] Abo-Elfetoh NM Farag AI Gabra RH. Impact of pain severity on functioning domains, sleep, and cognition in painful diabetic peripheral polyneuropathy patients. Middle East Curr Psychiatry. (2022) 29:83. doi: 10.1186/s43045-022-00243-8

[22] Croosu SS Gjela M Røikjer J Hansen TM Mørch CD Frøkjær JB. Cognitive function in individuals with and without painful and painless diabetic polyneuropathy-A cross-sectional study in type 1 diabetes. Endocrinology Diabetes Metab. (2023) 6:e420. doi: 10.1002/edm2.420, PMID: 37073434 PMC10335611

[23] Nguyen ML Wong D Barson E Staunton E Fisher CA. Cognitive dysfunction in diabetes-related foot complications: A cohort study. J Diabetes Metab Disord. (2024) 23:1017–38. doi: 10.1007/s40200-023-01381-4, PMID: 38932904 PMC11196439

[24] Yu X He H Wen J Xu X Ruan Z Hu R. Diabetes-related cognitive impairment: Mechanisms, symptoms, and treatments. Open Med (Warsaw Poland). (2025) 20:20241091. doi: 10.1515/med-2024-1091, PMID: 39822993 PMC11737369

[25] Milstein JL Ferris HA. The brain as an insulin-sensitive metabolic organ. Mol Metab. (2021) 52:101234. doi: 10.1016/j.molmet.2021.101234, PMID: 33845179 PMC8513144

[26] Cetinkalp S Simsir IY Ertek S. Insulin resistance in brain and possible therapeutic approaches. Curr Vasc Pharmacol. (2014) 12:553–64. doi: 10.2174/1570161112999140206130426, PMID: 23627981

[27] Luo JS Ning JQ Chen ZY Li WJ Zhou RL Yan RY. The role of mitochondrial quality control in cognitive dysfunction in diabetes. Neurochemical Res. (2022) 47:2158–72. doi: 10.1007/s11064-022-03631-y, PMID: 35661963 PMC9352619

[28] Grillo CA Woodruff JL Macht VA Reagan LP. Insulin resistance and hippocampal dysfunction: Disentangling peripheral and brain causes from consequences. Exp Neurol. (2019) 318:71–7. doi: 10.1016/j.expneurol.2019.04.012, PMID: 31028829

[29] Monteggia LM Barrot M Powell CM Berton O Galanis V Gemelli T. Essential role of brain-derived neurotrophic factor in adult hippocampal function. Proc Natl Acad Sci U S A. (2004) 101:10827–32. doi: 10.1073/pnas.0402141101, PMID: 15249684 PMC490019

[30] Willette AA Bendlin BB Starks EJ Birdsill AC Johnson SC Christian BT. Association of insulin resistance with cerebral glucose uptake in late middle-aged adults at risk for Alzheimer disease. JAMA Neurol. (2015) 72:1013–20. doi: 10.1001/jamaneurol.2015.0613, PMID: 26214150 PMC4570876

[31] Kim E Tolhurst AT Cho S. Deregulation of inflammatory response in the diabetic condition is associated with increased ischemic brain injury. J Neuroinflamm. (2014) 11:83. doi: 10.1186/1742-2094-11-83, PMID: 24886035 PMC4017808

[32] Muriach M Flores-Bellver M Romero FJ Barcia JM. Diabetes and the brain: oxidative stress, inflammation, and autophagy. Oxid Med Cell Longevity. (2014) 2014:102158. doi: 10.1155/2014/102158, PMID: 25215171 PMC4158559

[33] Gerstein HC Smith EE Ramasundarahettige C Desai D Awadalla P Broet P. Diabetes, brain infarcts, cognition, and small vessels in the Canadian alliance for healthy hearts and minds study. J Clin Endocrinol Metab. (2021) 106:e891–e8. doi: 10.1210/clinem/dgaa815, PMID: 33165530 PMC7823245

[34] Mechanisms of cognitive decline in newly diagnosed diabetics: A review of pathophysiological contributions and intervention strategies. MEDS Basic Med. (2024) 2(2). doi: 10.23977/medbm.2024.020203

[35] Chandran R He L Nie X Voltin J Jamil S Doueiry C. Magnetic resonance imaging reveals microemboli-mediated pathological changes in brain microstructure in diabetic rats: relevance to vascular cognitive impairment/dementia. Clin Sci (London England: 1979). (2022) 136:1555–70. doi: 10.1042/cs20220465, PMID: 36314470 PMC10066787

[36] Umegaki H. Neurodegeneration in diabetes mellitus. Adv Exp Med Biol. (2012) 724:258–65. doi: 10.1007/978-1-4614-0653-2_19, PMID: 22411248

[37] Sandireddy R Yerra VG Areti A Komirishetty P Kumar A. Neuroinflammation and oxidative stress in diabetic neuropathy: futuristic strategies based on these targets. Int J Endocrinol. (2014) 2014:674987. doi: 10.1155/2014/674987, PMID: 24883061 PMC4021687

[38] Kumar P Raman T Swain MM Mishra R Pal A. Hyperglycemia-induced oxidative-nitrosative stress induces inflammation and neurodegeneration via augmented tuberous sclerosis complex-2 (TSC-2) activation in neuronal cells. Mol Neurobiol. (2017) 54:238–54. doi: 10.1007/s12035-015-9667-3, PMID: 26738854

[39] Schmeichel AM Schmelzer JD Low PA. Oxidative injury and apoptosis of dorsal root ganglion neurons in chronic experimental diabetic neuropathy. Diabetes. (2003) 52:165–71. doi: 10.2337/diabetes.52.1.165, PMID: 12502508

[40] Kale MB Bhondge HM Wankhede NL Shende PV Thanekaer RP Aglawe MM. Navigating the intersection: Diabetes and Alzheimer's intertwined relationship. Ageing Res Rev. (2024) 100:102415. doi: 10.1016/j.arr.2024.102415, PMID: 39002642

[41] Nasrolahi A Mahmoudi J Noori-Zadeh A Haghani K Bakhtiyari S Darabi S. Shared pathological mechanisms between diabetes mellitus and neurodegenerative diseases. Curr Pharmacol Rep. (2019) 5:219–31. doi: 10.1007/s40495-019-00191-8

[42] Szablewski L. Associations between diabetes mellitus and neurodegenerative diseases. Int J Mol Sci. (2025) 26(2):542. doi: 10.3390/ijms26020542, PMID: 39859258 PMC11765393

[43] Ahmad R Chowdhury K Kumar S Irfan M Reddy GS Akter F. Diabetes mellitus: A path to amnesia, personality, and behavior change. Biology. (2022) 11(3):382. doi: 10.3390/biology11030382, PMID: 35336756 PMC8945557

[44] H S T T Vellapandian C. Gut-brain axis: unveiling the interplay between diabetes mellitus and Alzheimer's disease. Cureus. (2024) 16:e68083. doi: 10.7759/cureus.68083, PMID: 39347125 PMC11438540

[45] Bi T Zhang L Zhan L Feng R Zhao T Ren W. Integrated analyses of microbiomics and metabolomics explore the effect of gut microbiota transplantation on diabetes-associated cognitive decline in Zucker diabetic fatty rats. Front Aging Neurosci. (2022) 14:913002. doi: 10.3389/fnagi.2022.913002, PMID: 35721013 PMC9204715

[46] Zhang Q Jin K Chen B Liu R Cheng S Zhang Y. Overnutrition induced cognitive impairment: insulin resistance, gut-brain axis, and neuroinflammation. Front Neurosci. (2022) 16:884579. doi: 10.3389/fnins.2022.884579, PMID: 35873818 PMC9298971

[47] Bozanic A Toro P Bello-Lepe S Hurtado-Oliva J Beyle C Valdés C. Cognitive impairment with Type 2 Diabetes Mellitus among community-dwelling older adults in Chile: Prevalence, risk factors and cognitive characteristics. Front Hum Neurosci. (2022) 16:1070611. doi: 10.3389/fnhum.2022.1070611, PMID: 36741779 PMC9892451

[48] Wu X Tang Y He Y Wang Q Wang Y Qin X. Prevalence of cognitive impairment and its related factors among Chinese older adults: an analysis based on the 2018 CHARLS data. Front Public Health. (2024) 12:1500172. doi: 10.3389/fpubh.2024.1500172, PMID: 39776486 PMC11703964

[49] Moran C Gilsanz P Beeri MS Whitmer RA Lacy ME. Sex, diabetes status and cognition: findings from the study of longevity in diabetes. BMJ Open Diabetes Res Care. (2021) 9:e001646. doi: 10.1136/bmjdrc-2020-001646, PMID: 33509934 PMC7845709

[50] Jokisch M Platzbecker AL Gronewold J Jöckel KH Moebus S Erbel R. Gender- and age-specific association of known type 2 diabetes mellitus on incident mild cognitive impairment five years later: Results from the population-based Heinz Nixdorf Recall Study. Alzheimer's Dementia. (2024) 20:e084652. doi: 10.1002/alz.084652, PMID: 40502925 PMC12152370

[51] Reinke C. The effect of diabetes in the multifaceted relationship between education and cognitive function. BMC Public Health. (2024) 24:2584. doi: 10.1186/s12889-024-20156-x, PMID: 39334040 PMC11429487

[52] Peña-González P Mondragón-Maya A Silva-Pereyra J Roa-Rojas P. Cognitive reserve and executive functions in adults with type 2 diabetes. J Diabetes Res. (2020) 2020:7941543. doi: 10.1155/2020/7941543, PMID: 33083496 PMC7557905

[53] Connors MH Seeher K Teixeira-Pinto A Woodward M Ames D Brodaty H. Dementia and caregiver burden: A three-year longitudinal study. Int J geriatric Psychiatry. (2020) 35:250–8. doi: 10.1002/gps.5244, PMID: 31821606

[54] Saldana SL Guarnaccia CA. Comparing cognitive function in white Mexican & non-Hispanic white Americans with/without diabetes. J Diabetes Metab Disord. (2022) 21:599–605. doi: 10.1007/s40200-022-01022-2, PMID: 35673415 PMC9167396

[55] Lin Y Gong Z Ma C Wang Z Wang K. Relationship between glycemic control and cognitive impairment: A systematic review and meta-analysis. Front Aging Neurosci. (2023) 15:1126183. doi: 10.3389/fnagi.2023.1126183, PMID: 36776436 PMC9909073

[56] Herath PM Cherbuin N Eramudugolla R Anstey KJ. The effect of diabetes medication on cognitive function: evidence from the PATH through life study. BioMed Res Int. (2016) 2016:7208429. doi: 10.1155/2016/7208429, PMID: 27195294 PMC4853928

[57] Chapman LE Darling AL Brown JE. Association between metformin and vitamin B(12) deficiency in patients with type 2 diabetes: A systematic review and meta-analysis. Diabetes Metab. (2016) 42:316–27. doi: 10.1016/j.diabet.2016.03.008, PMID: 27130885

[58] Moore E Mander A Ames D Carne R Sanders K Watters D. Cognitive impairment and vitamin B12: a review. Int psychogeriatrics. (2012) 24:541–56. doi: 10.1017/s1041610211002511, PMID: 22221769

[59] Campbell JM Stephenson MD De Courten B Chapman I Bellman SM Aromataris E. Metformin use associated with reduced risk of dementia in patients with diabetes: A systematic review and meta-analysis. J Alzheimer's disease: JAD. (2018) 65:1225–36. doi: 10.3233/jad-180263, PMID: 30149446 PMC6218120

[60] Siddeeque N Hussein MH Abdelmaksoud A Bishop J Attia AS Elshazli RM. Neuroprotective effects of GLP-1 receptor agonists in neurodegenerative Disorders: A Large-Scale Propensity-Matched cohort study. Int Immunopharmacol. (2024) 143:113537. doi: 10.1016/j.intimp.2024.113537, PMID: 39486172

[61] Luan S Cheng W Wang C Gong J Zhou J. Impact of glucagon-like peptide 1 analogs on cognitive function among patients with type 2 diabetes mellitus: A systematic review and meta-analysis. Front Endocrinol. (2022) 13:1047883. doi: 10.3389/fendo.2022.1047883, PMID: 36387915 PMC9650490

[62] Vadini F Simeone PG Boccatonda A Guagnano MT Liani R Tripaldi R. Liraglutide improves memory in obese patients with prediabetes or early type 2 diabetes: a randomized, controlled study. Int J Obes (2005). (2020) 44:1254–63. doi: 10.1038/s41366-020-0535-5, PMID: 31965072

[63] Hansen HH Fabricius K Barkholt P Niehoff ML Morley JE Jelsing J. The GLP-1 receptor agonist liraglutide improves memory function and increases hippocampal CA1 neuronal numbers in a senescence-accelerated mouse model of Alzheimer's disease. J Alzheimer's disease: JAD. (2015) 46:877–88. doi: 10.3233/jad-143090, PMID: 25869785 PMC4878312

[64] Zhang Y Liao X Xu J Yin J Li S Li M. The promising potency of sodium-glucose cotransporter 2 inhibitors in the prevention of and as treatment for cognitive impairment among type 2 diabetes patients. Biomedicines. (2024) 12(12):2783. doi: 10.3390/biomedicines12122783, PMID: 39767690 PMC11673520

[65] Gui Z Wang J Zhang Y. Dapagliflozin improves diabetic cognitive impairment via indirectly modulating the mitochondria homeostasis of hippocampus in diabetic mice. BioFactors (Oxford England). (2024) 50:145–60. doi: 10.1002/biof.1998, PMID: 37596888

[66] Youn YJ Kim S Jeong HJ Ah YM Yu YM. Sodium-glucose cotransporter-2 inhibitors and their potential role in dementia onset and cognitive function in patients with diabetes mellitus: a systematic review and meta-analysis. Front Neuroendocrinol. (2024) 73:101131. doi: 10.1016/j.yfrne.2024.101131, PMID: 38367940

[67] Pan J Yang H Lu J Chen L Wen T Zhao S. The impact of SGLT2 inhibitors on dementia onset in patients with type 2 diabetes: A meta-analysis of cohort studies. Neuroendocrinology. (2025) 115(3-4):351–9. doi: 10.1159/000543533, PMID: 39799939

[68] Lardaro A Quarta L Pagnotta S Sodero G Mariani S Del Ben M. Impact of sodium glucose cotransporter 2 inhibitors (SGLT2i) therapy on dementia and cognitive decline. Biomedicines. (2024) 12(8):1750. doi: 10.3390/biomedicines12081750, PMID: 39200215 PMC11351143

[69] Gault VA Lennox R Flatt PR. Sitagliptin, a dipeptidyl peptidase-4 inhibitor, improves recognition memory, oxidative stress and hippocampal neurogenesis and upregulates key genes involved in cognitive decline. Diabetes Obes Metab. (2015) 17:403–13. doi: 10.1111/dom.12432, PMID: 25580570

[70] Yuan Y Zhang Y Lei M Guo X Yang X Ouyang C. Effects of DPP4 inhibitors as neuroprotective drug on cognitive impairment in patients with type 2 diabetes mellitus: A meta-analysis and systematic review. Int J Endocrinol. (2024) 2024:9294113. doi: 10.1155/2024/9294113, PMID: 38379936 PMC10878760

[71] Hu Y Xu J Wang J Zhu L Wang J Zhang Q. DPP-4 inhibitors suppress tau phosphorylation and promote neuron autophagy through the AMPK/mTOR pathway to ameliorate cognitive dysfunction in diabetic mellitus. ACS Chem Neurosci. (2023) 14:3335–46. doi: 10.1021/acschemneuro.2c00733, PMID: 37655714

[72] Meng J Yan R Zhang C Bai X Yang X Yang Y. Dipeptidyl peptidase-4 inhibitors alleviate cognitive dysfunction in type 2 diabetes mellitus. Lipids Health Dis. (2023) 22:219. doi: 10.1186/s12944-023-01985-y, PMID: 38082288 PMC10712048

[73] Rizzo MR Barbieri M Boccardi V Angellotti E Marfella R Paolisso G. Dipeptidyl peptidase-4 inhibitors have protective effect on cognitive impairment in aged diabetic patients with mild cognitive impairment. journals gerontology Ser A Biol Sci Med Sci. (2014) 69:1122–31. doi: 10.1093/gerona/glu032, PMID: 24671867

[74] Pintana H Apaijai N Chattipakorn N Chattipakorn SC. DPP-4 inhibitors improve cognition and brain mitochondrial function of insulin-resistant rats. J Endocrinol. (2013) 218:1–11. doi: 10.1530/joe-12-0521, PMID: 23591914

[75] Anirudhan A Ahmad SF Emran TB Angulo-Bejarano PI Sharma A Ahmed S. Comparative efficacy of metformin and glimepiride in modulating pharmacological network to increase BDNF levels and benefit type 2 diabetes-related cognitive impairment. Biomedicines. (2023) 11(11):2939. doi: 10.3390/biomedicines11112939, PMID: 38001940 PMC10669717

[76] Rosado AF Rosa PB Platt N Pierone BC Neis VB Severo Rodrigues AL. Glibenclamide treatment prevents depressive-like behavior and memory impairment induced by chronic unpredictable stress in female mice. Behav Pharmacol. (2021) 32:170–81. doi: 10.1097/fbp.0000000000000599, PMID: 33079735

[77] Wu CY Iskander C Wang C Xiong LY Shah BR Edwards JD. Association of sulfonylureas with the risk of dementia: A population-based cohort study. J Am Geriatrics Soc. (2023) 71:3059–70. doi: 10.1111/jgs.18397, PMID: 37218376

[78] Tian S Jiang J Wang J Zhang Z Miao Y Ji X. Comparison on cognitive outcomes of antidiabetic agents for type 2 diabetes: A systematic review and network meta-analysis. Diabetes/metabolism Res Rev. (2023) 39:e3673. doi: 10.1002/dmrr.3673, PMID: 37302139

[79] Vandersmissen J Dewachter I Cuypers K Hansen D. The impact of exercise training on the brain and cognition in T2DM, and its physiological mediators: a systematic review. medRxiv. (2024) 11:42. doi: 10.1101/2024.09.19.24313875, PMID: 40274715 PMC12022206

[80] Sun Z Liu H Yan M Zeng H Hu Y Tian X. The effect of multi-component exercise on cognition function in patients with diabetes: A systematic review and meta-analysis. PLoS One. (2024) 19:e0304795. doi: 10.1371/journal.pone.0304795, PMID: 38900771 PMC11189216

[81] Cooke S Pennington K Jones A Bridle C Smith MF Curtis F. Effects of exercise, cognitive, and dual-task interventions on cognition in type 2 diabetes mellitus: A systematic review and meta-analysis. PLoS One. (2020) 15:e0232958. doi: 10.1371/journal.pone.0232958, PMID: 32407347 PMC7224461

[82] Wang R Yan W Du M Tao L Liu J. The effect of physical activity interventions on cognition function in patients with diabetes: A systematic review and meta-analysis. Diabetes/metabolism Res Rev. (2021) 37:e3443. doi: 10.1002/dmrr.3443, PMID: 33616310 PMC8519002

[83] Cuevas HE Stuifbergen AK Brown SA Ward C. A. A nurse-led cognitive training intervention for individuals with type 2 diabetes. Res gerontological Nurs. (2019) 12:203–12. doi: 10.3928/19404921-20190612-01, PMID: 31335962 PMC8573732

[84] Dyer AH Briggs R Mockler D Gibney J Kennelly SP. Non-pharmacological interventions for cognition in patients with Type 2 diabetes mellitus: a systematic review. QJM: monthly J Assoc Physicians. (2020) 113:155–61. doi: 10.1093/qjmed/hcz053, PMID: 30825309

[85] Goetzl EJ Mustapic M Kapogiannis D Eitan E Lobach IV Goetzl L. Cargo proteins of plasma astrocyte-derived exosomes in Alzheimer's disease. FASEB J. (2016) 30:3853–9. doi: 10.1096/fj.201600756R, PMID: 27511944 PMC5067254

[86] Chi H Yao R Sun C Leng B Shen T Wang T. Blood neuroexosomal mitochondrial proteins predict Alzheimer disease in diabetes. Diabetes. (2022) 71:1313–23. doi: 10.2337/db21-0969, PMID: 35287177

[87] Kumar A Cleary JA Su Y Singh S Howard M Hayden KM. Multi-omics analysis of neuron-derived small extracellular vesicles to identify novel peripheral biomarker of cognitive impairment in individuals with type 2 diabetes. Alzheimer's Dementia. (2024) 20:e091878. doi: 10.1002/alz.091878

[88] Van Den Berg MMJ Krauskopf J Ramaekers JG Kleinjans JCS Prickaerts J Briede JJ. Circulating microRNAs as potential biomarkers for psychiatric and neurodegenerative disorders. Prog Neurobiol. (2020) 185:101732. doi: 10.1016/j.pneurobio.2019.101732, PMID: 31816349

[89] Liu X Zhao Z Chen D Zhang Z Lin X Shen Z. SIRT1 and miR-34a-5p expression in PBMCs as potential biomarkers for patients with type 2 diabetes with cognitive impairments. J Clin Endocrinol Metab. (2024) 109:815–26. doi: 10.1210/clinem/dgad562, PMID: 37758217

[90] Chen MD Deng CF Chen PF Li A Wu HZ Ouyang F. Non-invasive metabolic biomarkers in initial cognitive impairment in patients with diabetes: A systematic review and meta-analysis. Diabetes Obes Metab. (2024) 26:5519–36. doi: 10.1111/dom.15916, PMID: 39233493

[91] Ehtewish H Arredouani A El-Agnaf O. Diagnostic, prognostic, and mechanistic biomarkers of diabetes mellitus-associated cognitive decline. Int J Mol Sci. (2022) 23(11):6144. doi: 10.3390/ijms23116144, PMID: 35682821 PMC9181591

[92] Zhang T Shaw M Cherbuin N. Association between type 2 diabetes mellitus and brain atrophy: A meta-analysis. Diabetes Metab J. (2022) 46:781–802. doi: 10.4093/dmj.2021.0189, PMID: 35255549 PMC9532183

[93] Zhang W Gao C Qing Z Zhang Z Bi Y Zeng W. Hippocampal subfields atrophy contribute more to cognitive impairment in middle-aged patients with type 2 diabetes rather than microvascular lesions. Acta Diabetologica. (2021) 58:1023–33. doi: 10.1007/s00592-020-01670-x, PMID: 33751221

[94] Naidu VV Ismail K Amiel S Kohli R Crosby-Nwaobi R Sivaprasad S. Associations between retinal markers of microvascular disease and cognitive impairment in newly diagnosed type 2 diabetes mellitus: A case control study. PLoS One. (2016) 11:e0147160. doi: 10.1371/journal.pone.0147160, PMID: 26771382 PMC4714814

[95] Doney ASF Nar A Huang Y Trucco E MacGillivray T Connelly P. Retinal vascular measures from diabetes retinal screening photographs and risk of incident dementia in type 2 diabetes: A GoDARTS study. Front digital Health. (2022) 4:945276. doi: 10.3389/fdgth.2022.945276, PMID: 36120710 PMC9470757

[96] Pedersen FN Stokholm L Lois N Yang D Cheung CY Biessels GJ. Structural and metabolic retinal changes associated with mild cognitive impairment in type 2 diabetes. Diabetes. (2023) 72:1853–63. doi: 10.2337/db23-0025, PMID: 37725903

[97] Majimbi M McLenachan S Nesbit M Chen FK Lam V Mamo J. *In vivo* retinal imaging is associated with cognitive decline, blood-brain barrier disruption and neuroinflammation in type 2 diabetic mice. Front Endocrinol. (2023) 14:2023.1224418. doi: 10.3389/fendo.2023.1224418, PMID: 37850093 PMC10577437

